# Transcriptomics‐based analysis of the causes of sugar receding in Feizixiao litchi (*Litchi chinensis* Sonn.) pulp

**DOI:** 10.3389/fpls.2022.1083753

**Published:** 2022-12-22

**Authors:** Junjie Peng, Jingjia Du, Ma Wuqiang, Tiantian Chen, Xian Shui, Haizhi Liao, Xiaokai Lin, Kaibing Zhou

**Affiliations:** ^1^ Sanya Nanfan Research Institute of Hainan University, Sanya, China; ^2^ College of Horticulture, Hainan University, Haikou, China

**Keywords:** glycolytic pathway, litchi, phosphofructokinase, sugar receding, transcriptome sequencing

## Abstract

To investigate the causes of the “sugar receding” in ‘Feizixiao’ litchi (*Litchi chinensis* Sonn.) pulp, the main sugar contents and sucrose metabolism enzyme activities were measured in pulp obtained in 2020 and 2021. Pulp RNA obtained in 2020 was extracted at 35, 63, and 69 days after anthesis (DAA) for transcriptome sequencing analysis. The differential expression of genes was verified by real-time PCR for both years. The results showed that after 63 DAA, the contents of soluble sugars and sucrose decreased, and the contents of fructose and glucose increased in both years. The dynamic changes in sucrose metabolism enzyme activities were similar in both years. After 63 DAA, except for acid invertase (AI) in 2021, the activities of other enzymes decreased significantly, and the net activity of sucrose metabolism enzymes showed a strong sucrose cleavage activity. There were 18061, 19575, and 985 differentially expressed genes in 35 d vs. 63 d, 35 d vs. 69 d, and 63 d vs. 69 d, respectively. Ninety-one sugar metabolism genes were screened out, including sucrose synthase (SS), sucrose phosphate synthase (SPS), AI, neutral invertase (NI), hexokinase (HK), glucose 6-phosphate dehydrogenase (G6PD), 6-phosphogluconate dehydrogenase (6PGD), phosphofructokinase (PFK), and pyruvate kinase (PK) genes. In 63 d vs. 69 d, seventy-five percent of sucrose metabolism genes were downregulated, seventy-seven percent of genes in glycolysis (EMP) were upregulated and the PFK genes were significantly upregulated. There was a significant linear correlation between the expression of 15 genes detected by real-time PCR and the transcriptome sequencing results (*r_2020_* = 0.9139, *r_2021_* = 0.8912). These results suggest that the upregulated expression of PFK genes at maturity may enhance PFK activity and promote the degradation of soluble sugar in pulp through the EMP pathway, resulting in decreased soluble sugar and sucrose contents and “sugar receding” in pulp. Moreover, the downregulated expression of sucrose metabolism genes in pulp decreased the activities of these enzymes, but the net activity of these enzymes resulted in cleaved sucrose and replenished levels of reducing sugars, resulting in a stable reducing sugar content.

## 1 Introduction

Litchi (*Litchi chinensis* Sonn.) is an evergreen fruit tree belonging to the Sapindaceae family, native to China (Menzel, 2001) and cultivated in the United States, Australia, Brazil, India, and Thailand and other places (Pareek, 2016). It is popular among consumers for its sweet and sour taste (Jiang et al., 2006). ‘Feizixiao’ litchi is one of the main cultivars in Hainan Province, and is becoming increasingly popular in the world consumer market (Jiang et al., 2012). It has been shown that the pulp of the fruit undergoes “sugar receding” when fruit ripens (Wang et al., 2017), adversely affecting its quality, which is not conducive to maximizing its economic benefits. Therefore, investigating the physiological and molecular mechanisms of the “sugar receding” phenomenon is beneficial for artificially regulating the fruit sugar contents and improving the commercial value of ‘Feizixiao’ litchi.

Different varieties of litchi accumulate different types of sugars. ‘Feizixiao’ litchi pulp mainly contains the reducing sugars (fructose and glucose) and sucrose (Wang et al., 2006). Leaf photosynthetic products undergoing long-distance transport are mainly in the form of sucrose from source tissues to sink tissues (Braun et al., 2014), sucrose metabolism enzymes affect the accumulation of sucrose in fruit (Koch, 2004). Sucrose metabolism enzymes include sucrose synthase (SS), sucrose phosphate synthase (SPS), and invertases (INVs). INVs are divided into acid invertase (AI) and neutral invertase (NI). AI is found in the vacuole, cell wall and between cell walls, and NI is found in the cytoplasm (Yang et al., 2013). Moreover, SS catalyzes the reversible reaction between fructose, uridine diphosphate glucose and sucrose (Kaur and Das, 2022), and SPS is the key rate-limiting enzyme that regulates the catalyzing process from fructose-6-phosphate to sucrose (Sun et al., 2020), while INVs catalyze the cleavage of sucrose to produce fructose and glucose (Ruan et al., 2010). These enzymes regulate the conversion between sucrose and reducing sugars to control the composition and content of soluble sugars imported and stored in fruits (Ren et al., 2021). For example, A previous study on peaches (*Prunus persica* L.) showed that ABA treatment increased the activities of SS and SPS, and decreased the activity of AI, resulting in increased sucrose content in ABA-treated fruit (Zhao et al., 2022). In a study on strawberries (*Fragaria* x *ananassa*), SS activity was low and had no significant contribution to sink strength, and INV activity was high, which are the main enzymes of sucrose cleavage (Basson et al., 2010). Moreover, sucrose transport related proteins affect the transport and transfer of sucrose (Milne et al., 2018), including sucrose transporters (SUTs) and sugars will eventually be exported transporters (SWEETs), which are also related to sugar accumulation. For example, the apple (*Malus domestica*) sucrose transporter MdSUT2.2 is highly expressed in ripening fruit, and the over­expression of *MdSUT2.2* increases sugar contents in apple (Ma et al., 2017). In the high-sucrose budding variety of ‘Nanguo (NG)’ pear (*Pyrus ussuriensis* Maxim.), *PuSWEET15* is more highly expressed in developing fruit, which accumulates more sucrose than ‘NG’ (Li et al., 2020a).

Low temperature can inhibit the rate of glycolysis (EMP) and lead to the accumulation of sugar (Trevanion and Kruger, 1991), which indicates that sugar content is also controlled by other pathways. The glycolysis-tricarboxylic acid cycle aerobic respiration pathway and pentose phosphate pathway (PPP), which are important downstream metabolic pathways of reducing sugars, use reducing sugars as substrates to generate various intermediate substances and energy for normal fruit growth and development (Kou et al., 2018). EMP is the first stage of the aerobic respiration pathway of the glycolysis-tricarboxylic acid cycle, so the decrease in soluble sugar content in ‘Feizixiao’ litchi pulp in the late stage of growth and development should be related to EMP and PPP. The key enzymes in EMP are hexokinase (HK), phosphofructokinase (PFK) and pyruvate kinase (PK) (Zhang et al., 2018); the key enzymes in PPP are glucose-6-phosphate dehydrogenase (G6PD) (Castiglia et al., 2015; Ramos-Martinez, 2017) and 6-phosphogluconate dehydrogenase (6PGD) (Li et al., 2020b). HK catalyzes the phosphorylation of reducing sugars, which is the first important reaction of hexose metabolism (Kandel-Kfir et al., 2006). Only two hexose phosphorylases, fructokinase (FK) and HK, have been discovered thus far (Granot et al., 2013), while glucose is converted into glucose 6-phosphate under the catalysis of HK to enter EMP and PPP, so HK affects the metabolism of both EMP and PPP rates. Fructose is converted to fructose 6-phosphate by FK and HK, and then fructose 6-phosphate enters EMP and PPP (Pego and Smeekens, 2000).

In this study, the sugar contents of ‘Feizixiao’ litchi pulp and the activities of sucrose metabolism enzymes were measured in two separate years. Through transcriptome sequencing, the differentially expressed genes of ‘Feizixiao’ litchi pulp at different developmental stages were compared. We identified the key sugar metabolism genes that lead to the “sugar receding” of ‘Feizixiao’ litchi, used real-time PCR to verify the expression patterns of these genes, and analyzed the causes of “sugar receding” in ‘Feizixiao’ litchi pulp, which will provide a theoretical basis for artificially adjusting the sugar contents of ‘Feizixiao’ litchi pulp.

## 2 Materials and methods

### 2.1 Plant materials

At team 5 in Jingpai Village, Chengmai County, Hainan Province, five 16-year-old ‘Feizixiao’ litchi trees with uniform growth potential and no clear pest or health issues were selected for the experiment. ‘Feizixiao’ litchi from the garden entered the physiological fruit drop period in early April, the fruit expansion period in late April, and the fruit maturity period in mid-May (Liao et al., 2022). Sampling was performed during the fruit expansion period and fruit ripening period, that is, starting from 35 days after anthesis (DAA) (19, Apr, 2020, 18, Apr, 2021), and five fruit of the same size in the middle and periphery of each tree were selected. The size and coloring condition of the five fruits were used as the reference for each sampling. Highly similar fruit were selected as the materials, and thirty fruits were taken from each tree. The sampling times were 35, 42, 50, 56, 63, and 69 DAA in 2020 and 35, 42, 49, 56, 63, and 70 DAA in 2021. After harvesting the samples, they were placed in liquid nitrogen for quick freezing, and then taken back to the laboratory and stored in an ultralow temperature freezer (-80°C).

### 2.2 Extraction and determination of fructose, glucose and sucrose

The method referenced in Wang et al. (2006) was performed with some modifications. We weighed 0.5 g of pulp in a mortar, heated it in a microwave oven for 30 s, added 5 mL of 90% ethanol, ground the sample thoroughly, centrifuged it at 10,000 *g*_n_ for 15 min, aspirated the supernatant and added 5 mL of 90% ethanol for another extraction. Then, we combined the two supernatants, which were evaporated in a 90°C water bath, Then, the remaining sample was fixed with 10 mL of deionized water, and a small amount was aspirated with a syringe and filtered through a 0.45 μm membrane for testing. The sugar contents were determined on a Waters 2695 high-performance liquid chromatograph with an evaporative light scattering detector and a Boston Green Amino Column (4.6×250 mm, 5 μm). The mobile phase ratio was acetonitrile:water = 8:2, the flow rate was 1 mL min^-1^, the column temperature was 35°C, and the injection volume was 10 μL. High-purity glucose, fructose and sucrose (Beijing Tanmo Quality Inspection Technology Co., Ltd.) were used as the standards, and high-purity acetonitrile (Sinopharm Chemical Reagent Co., Ltd.) was used as the mobile phase. The sum of the fructose, glucose and sucrose contents was considered the total soluble sugar contents.

### 2.3 Determination of sucrose metabolism enzyme activities

The pulp sucrose synthase cleavage (SS-C) and synthesis (SS-S) and AI, NI, and SPS activities were measured by double antibody sandwich-enzyme-linked immunosorbent assay kits (Catalog Nos: KT8013-A, KT50452-A, KT5045-A, KT8107-A, KT5044-A, Jiangsu Kete Biotechnology Co., Ltd., Yancheng, Jiangsu, China) following the manufacturer’s protocol.

### 2.4 Transcriptome sequencing analysis and real-time PCR verification

#### 2.4.1 RNA extraction, library construction, sequencing and data filtering

According to the changes in the total soluble sugar content in ‘Feizixiao’ litchi pulp in 2020, RNA was extracted from the pulp at 35, 63, and 69 DAA for transcriptome sequencing, with 3 biological replicates in each time period. A plant total RNA extraction kit (RNAprep Pure Plant Plus Kit, catalog No: DP441) from Tiangen Biochemical Technology Co., Ltd. was used for RNA extraction. The procedure was carried out according to the instructions provided in the kit. RNA integrity and DNA contamination were analyzed by agarose gel electrophoresis. RNA purity, concentration, and integrity were accurately measured on a NanoPhotometer spectrophotometer, a Qubit 2.0 Fluorometer, and an Agilent 2100 Bioanalyzer, respectively. Then, a cDNA library was constructed from the high-quality RNA by Wuhan Metwell Biotechnology Co. Ltd. After the library was constructed, real-time PCR, a Qubit 2.0 fluorometer, and an Agilent 2100 bioanalyzer were used for quality inspection. Transcriptome sequencing was performed on the Illumina HiSeq platform after the library was qualified. Then, the raw reads were filtered to remove low-quality reads with adapters and to obtain clean reads.

#### 2.4.2 Sequence assembly

Trinity software was used to splice clean reads, and Corset (https://code.google.com/p/corset-project/) was used to perform hierarchical clustering according to the number of reads and expression patterns of the aligned transcripts. The obtained data were stored in FASTA format, and the longest transcript obtained after hierarchical clustering was used as a unigene.

#### 2.4.3 Gene annotation and differential gene screening

Unigenes were inputted into the Kyoto Encyclopedia of Genes and Genomes (KEGG), nr, Swiss-Prot, Gene Ontology (GO), Clusters of Orthologous Groups of proteins/Eukaryotic Orthologous Groups of proteins (COG/KOG), and TrEMBL databases using BLAST software, and the predicted amino acid sequences of the unigenes were compared to those in Pfam database using HMMER software to obtain annotation information. RSEM software was used for mapping, and fragments per kilobase of transcript per million fragments mapped (FPKM) was used to calculate the expression of the unigenes. DESeq2 software (Love et al., 2014; Varet et al., 2016) was used to screen the differentially expressed genes.

#### 2.4.4 Differential gene enrichment analysis

GO and KEGG significant enrichment analysis of differentially expressed genes takes GO terms and KEGG pathways as units, respectively, and apply a hypergeometric test to find the pathways and GO terms that are significantly enriched for differentially expressed genes in the context of the whole genome.

#### 2.4.5 Primer design and real-time PCR verification

Fifteen differentially expressed genes were selected, and real-time PCR primers, synthesized by Shanghai Bioengineering Co., Ltd., were designed with Prime3 (https://bioinfo.ut.ee/primer3-0.4.0/). The extracted pulp RNA was reverse transcribed into cDNA using a cDNA synthesis Kit from Vazyme Biotechnology Co., Ltd. (Nanjing, China) and a T100FM Thermal Cycler PCR instrument from BIO-RAD (USA). The experiment was performed according to the instructions provided in the kit. Real-time PCR verification was performed with Taq Pro Universal SYBR qPCR Master Mix (Vazyme Code: Q712-02) from Vazyme Biotechnology Co., Ltd. (Nanjing, China) and a qTOWER^3^ instrument (Jena, Germany). The relative expression of genes was calculated using the 2^−ΔΔCt^ method (Livak and Schmittgen, 2001) with 35 DAA as the reference, and litchi *actin* was used as the internal reference gene (Jiang et al., 2017). The sequences of the primers are shown in **Supplementary Table S1**.

### 2.5 Data analysis and graphing

Data statistics and graphing were performed with Excel, and data analysis was performed with SAS software, in which the ANOVA program was used for variance analysis and Duncan’s new multiple range test was used for multiple comparisons. The differential gene heatmap and Venn plot were drawn with TBtools software (Chen et al., 2020).

## 3 Results and analysis

### 3.1 Identification of soluble sugar components

The high-performance liquid chromatograms of the pulp sugar components of the samples harvested at 63 and 69 DAA in 2020 are shown in **Supplementary Figures S1A, B**. There was no significant change in fructose or glucose content and a significant decrease in sucrose content during the “sugar receding” period. The high-performance liquid chromatograms of the sugar content in the pulp at 63 and 70 DAA in 2021 are shown in **Supplementary Figures S1C, D** and the changes in the sugar content during the “sugar receding” period are consistent with those in 2020. The results from both years indicated that there was no significant change in reducing sugar content and a significant decrease in sucrose content in the “sugar receding” period.

### 3.2 Changes in the contents of fructose, glucose and sucrose in litchi pulp

**Figure 1:**
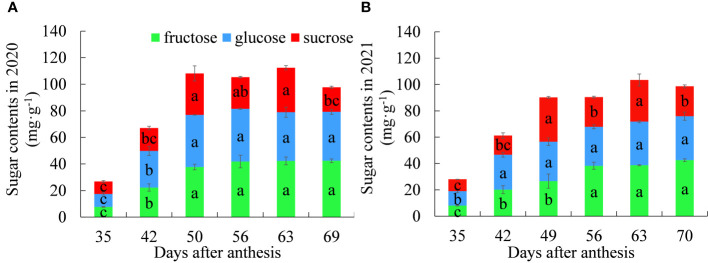
Dynamic changes in the contents of fructose, glucose and sucrose between **(A)** 2020 and **(B)** 2021. Different letters indicate significant differences during growth at *P* = 0.05. Error bars represent the standard error of the mean (*SEM*).

The dynamic changes in fructose, glucose and sucrose contents in litchi pulp over the two years are shown in **Figures 1A, B**. In 2020, the fructose content increased significantly from 35 to 50 DAA and became stable after 56 DAA. In 2021, the fructose content increased significantly from 35 to 56 DAA and then stabilized. The dynamics of fructose contents for both years were basically the same, indicating that pulp fructose continues to accumulate and increase with the expansion of the fruit and then stabilizes during maturity. Therefore, fructose is not the main component causing the “sugar receding” phenomenon during maturity.

In 2020, the glucose content increased significantly from 35 to 50 DAA and remained stable from 50 to 69 d with no significant difference between any two stages. In 2021, the glucose content increased significantly from 35 to 42 DAA and then stabilized. The dynamics of glucose contents for the two years were basically the same, indicating that glucose accumulates rapidly during the rapid expansion period of the fruit and tends to become stable during maturity. Therefore, glucose is not the main component causing the “sugar receding” phenomenon during maturity.

The sucrose content trends in both years were consistent, with a large amount of accumulation in the fruit expansion stage and a large amount of cleavage in the late stage of fruit ripening, indicating that sucrose is the main component causing the “sugar receding” phenomenon during maturity.

The dynamic changes in soluble sugar content in pulp showed the same trend. The soluble sugar contents all increased significantly in the early stage of fruit expansion, tended to become stable in the late stage of fruit expansion, peaked at 63 DAA, and then began to decline; then, the phenomenon of “sugar receding” occurred.

In summary, the content of reducing sugars continued to increase with fruit growth and development; the dynamic trends of sucrose and soluble sugar are highly similar, and their linear correlation is significant for two years (*r_2020_* = 0.8611, *r_2021_* = 0.8818), which indicates that the pulp of ‘Feizixiao’ litchi mainly accumulates reducing sugars, and the “sugar receding” phenomenon in the pulp is caused by the decrease in the sucrose content in the pulp.

### 3.3 Changes in the activities of sucrose metabolism enzymes

**Figure 2:**
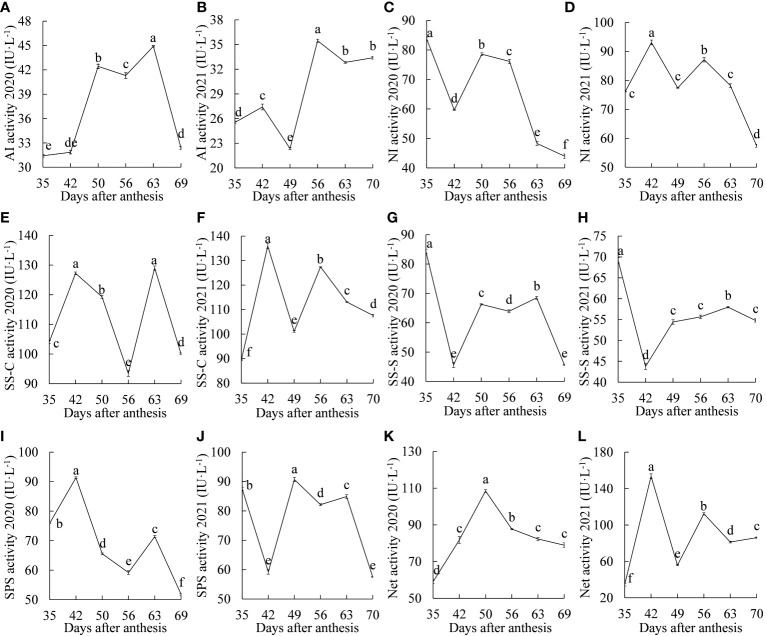
Dynamic changes in sucrose metabolism enzyme activities between 2020 and 2021. **(A, B)** acid invertase; **(C, D)** neutral invertase; **(E, F)** sucrose synthase-cleavage; **(G, H)** sucrose synthase-synthesis; **(I, J)** sucrose phosphate synthase; **(K, L)** the net activity of sucrose metabolism enzymes. Different letters indicate significant differences during growth at *P* = 0.05. Error bars represent the standard error of the mean (*SEM*).

The dynamic changes in AI activity are shown in **Figures 2A, B**. In 2020, the activity of AI increased significantly from 35 to 50 DAA, decreased significantly at 56 DAA, and then decreased significantly after reaching the maximum value at 63 DAA. In 2021, there was a rising-falling-rising-falling trend, the difference was significant between any two stages from 35 to 63 DAA, and there was no significant change after 63 DAA. The dynamic changes in AI activity in the two years were different, which may have been related to the different temperatures, amounts of light and other factors that occurred within the two years. However, in general, the overall activity of AI was low for both years, possibly indicating that AI had less of an effect on sucrose metabolism in ‘Feizixiao’ litchi pulp.

The dynamic changes in NI activity are shown in **Figures 2C, D**. In 2020, the activity of NI decreased, increased, and then decreased again, and the difference in activity between any two stages was significant. In 2021, NI activity increased and decreased twice, showing an “M”-type change trend, and reached maximum values at 35 d and 56 DAA, with a significant decline after 56 DAA. NI activity in both years showed a significant downward trend after 56 DAA.

The dynamic changes in SS-C activity are shown in **Figures 2E, F**. In 2020, the activity of SS-C showed an “M”-type change trend, reaching two peaks at 42 and 63 DAA. There was no significant difference between them, and there were significant differences in the rest of the stages, with significant declines from 42 to 56 DAA and 63 to 69 DAA. In 2021, there was also an “M”-type change trend, reaching two peaks at 42 and 52 DAA, with significant declines from 42 to 49 DAA and 56 to 70 DAA. The activity of SS-C decreased significantly in the “sugar receding” period in both years, but the activity was still higher than the maximum INV activity, which may indicate that the cleavage of sucrose is mainly catalyzed by SS.

The dynamic changes in SS-S activity are shown in **Figures 2G, H**. In 2020, the activity of SS-S showed a dynamic trend of inclines and declines, and the difference in activity between any two stages was significant. The enzyme activity decreased and then increased significantly from 35 to 49 DAA, reached the maximum value at 63 DAA and was significantly higher than that at the other three stages after 49 DAA. The dynamic trend of SS-S activity in the two years was basically the same, and SS-S activity in the “sugar receding” period decreased significantly.

The dynamic changes in SPS activity are shown in **Figures 2I, J**. In 2020, SPS activity showed a trend of a significant increase and then a significant decrease from 35 to 50 DAA. In 2021, it showed a trend of a significant decline and then a significant increase from 35 to 49 DAA. In the middle and late stages of fruit growth and development, there was a significant decrease, increase and then decrease trend in both years, and it decreased significantly in the “sugar receding” period.

The dynamic changes in the net activity of sucrose metabolism enzymes (difference between the cleavage and synthesis activities of sucrose metabolism enzymes) are shown in **Figures 2K, L**. In 2020, there was a unimodal trend, reaching a maximum value at 50 DAA, decreasing significantly at 56 DAA, and continuing to gradually decrease. In 2021, there was a rising-falling-rising-falling-rising trend with significant differences between any two stages. The net activity of sucrose metabolism enzymes for both years remained at a high level in the “sugar receding” period, which may have caused the significant decrease in sucrose content in the “sugar receding” period.

In summary, the dynamic changes in sucrose metabolism enzyme activities were similar for both years. In the “sugar receding” period, except for the activity of AI, which did not change significantly in 2021, the activities of the other enzymes decreased significantly. During the growth and development of ‘Feizixiao’ litchi pulp, the activity of SS-C was the highest among sucrose-cleaving enzymes, followed by NI, and that of AI was the lowest, which may indicate that SS plays a major role in cleaving sucrose. The activity of SS-C was consistently higher than that of SS-S, which may indicate that SS mainly cleaves sucrose.

### 3.4 Transcriptome sequencing results

As shown in **Table 1**, the transcriptome sequencing data were filtered to obtain an average of 43,676,652 clean reads and 6.55 Gb of clean base. The proportion of clean reads in each sample was greater than 96%, Q20 was above 98%, Q30 was above 94%, and the GC content was higher than 45%, indicating that the sequencing quality was favorable for subsequent analyses.

**Table 1:** Statistical results of the transcriptome sequencing of ‘Feizixiao’ litchi.

Sample	Raw Reads	Clean Reads	Clean Base(G)	Q20(%)	Q30(%)	GC Content(%)
35d-1 35d-2 35d-3	46,959,760 44,303,098 46,957,052	45,551,644 43,024,646 45,774,790	6.83 6.45 6.87	98.3 98.34 98.29	94.88 95 94.86	47.25 45.96 45.91
63d-1 63d-2 63d-3	45,892,812 46,358,114 45,275,344	44,640,602 45,171,552 44,009,558	6.7 6.78 6.6	98.46 98.33 98.34	95.21 94.93 94.93	45.06 45.38 45.28
69d-1 69d-2 69d-3	42,158,766 42,572,238 45,151,584	40,912,018 41,238,516 43,462,948	6.14 6.19 6.52	98.4 98.5 98.35	95.04 95.33 94.98	45.12 45.35 45.43
Mean	44,984,665	43,676,652	6.55	98.35	94.97	45.55
Total	539,815,982	524,119,832				

Q20: The percentage of the number of bases with a Qphred value no less than 20 in the total number of bases. Q30: The percentage of the number of bases with a Qphred value no less than 30 in the total number of bases. GC Content: The percentage of the sum of the quantities of G and C in the total number of bases in high-quality reads.

### 3.5 Screening results of differentially expressed genes

**Figure 3:**
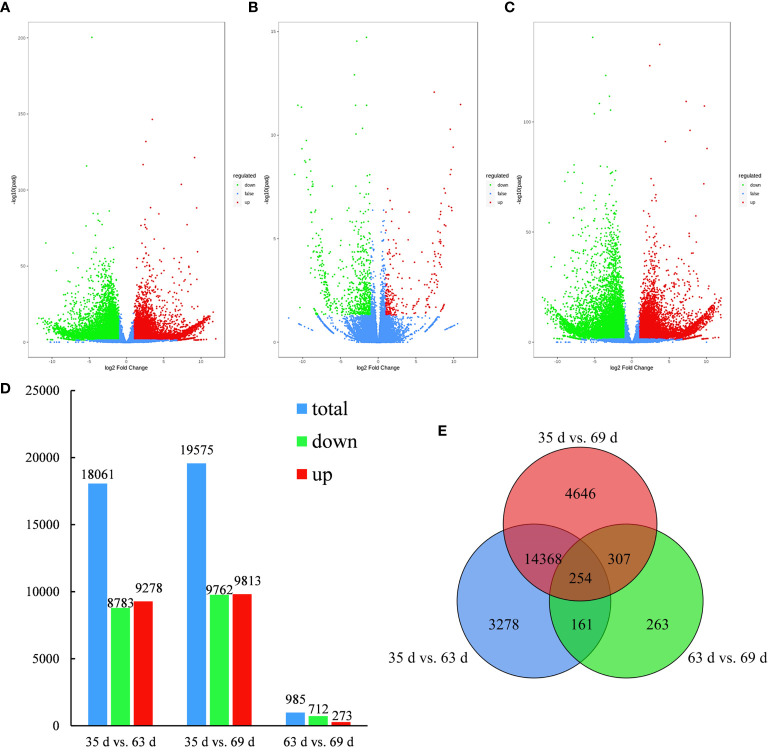
Volcano plot, bar plot and Venn diagram of differentially expressed genes. **(A–C)** The volcano plot of 35 d vs. 63 d, 63 d vs. 69 d, 35 d vs. 69 d. **(D)** Bar plot of differentially expressed genes generated by a comparison of 35 d vs. 63 d, 35 d vs. 69 d, 63 d vs. 69 d. **(E)** Venn diagram of differentially expressed genes generated by a comparison 35 d vs. 63 d, 35 d vs. 69 d, 63 d vs. 69 d.

As shown in **Figures 3A–D**, 18,061 genes were differentially expressed (8783 genes were downregulated, and 9278 genes were upregulated) in 35 d vs. 63 d; 19,575 genes were differentially expressed (9762 genes were downregulated, and 9813 genes were upregulated) in 35 d vs. 69 d; and 985 genes were differentially expressed (712 genes were downregulated, and 273 genes were upregulated) in 63 d vs. 69 d. **Figure 3E** shows that 254 differentially expressed genes were common to all three groups, and 263 differentially expressed genes were unique to 63 d vs. 69 d. In 35 d vs. 69 d, the number of differentially expressed genes was the highest, and the number of differentially expressed genes was the lowest in 63 d vs. 69 d. The number of differentially expressed genes between the two groups was significantly different, indicating that some genes are differentially expressed in the various fruit growth and development stages and that the number of differentially expressed genes increases as fruit growth and development progress.

### 3.6 GO enrichment analysis of differentially expressed genes

**Figure 4:**
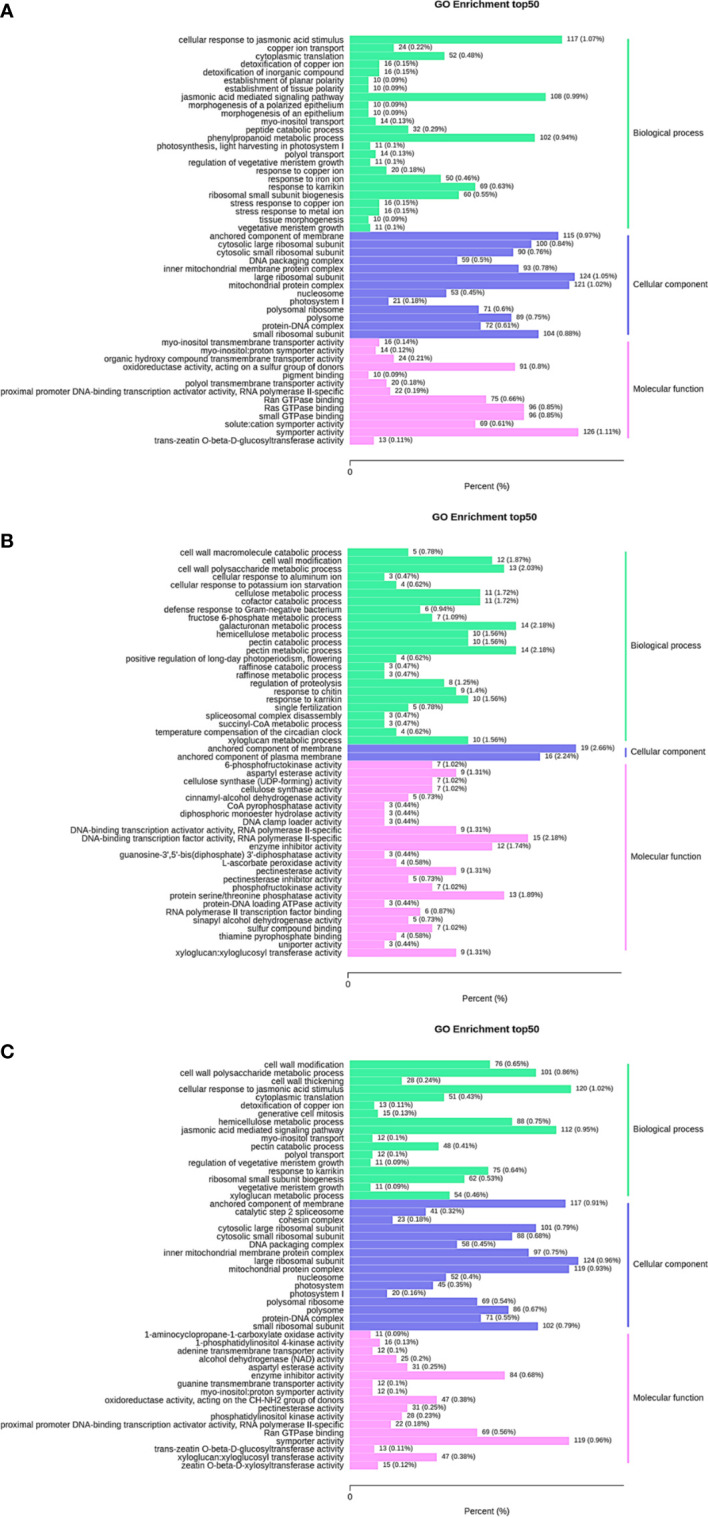
GO enrichment column plot. Differentially expressed genes generated by a comparison of **(A)** 35 d vs. 63 d, **(B)** 63 d vs. 69 d, **(C)** 35 d vs. 69 d are grouped into different GO terms of three ontologies: biological process, cellular component and molecular function.

GO enrichment analysis was performed on the differentially expressed genes in 35 d vs. 63 d, 63 d vs. 69 d, and 35 d vs. 69 d, and the 50 GO terms with the lowest q value were selected for representation. The results are shown in **Figures 4A–C**. Differentially expressed genes in all groups were divided into 3 major categories: biological process, cellular component and molecular function. The GO enrichment analysis results of differentially expressed genes in the three groups showed partial differences. Notably, seven PFK genes were enriched in the biological process and molecular function categories in 63 d vs. 69 d. It was speculated that the PFK genes influenced the changes in sugar content during the “sugar receding” period.

### 3.7 KEGG enrichment analysis of differentially expressed genes

**Figure 5:**
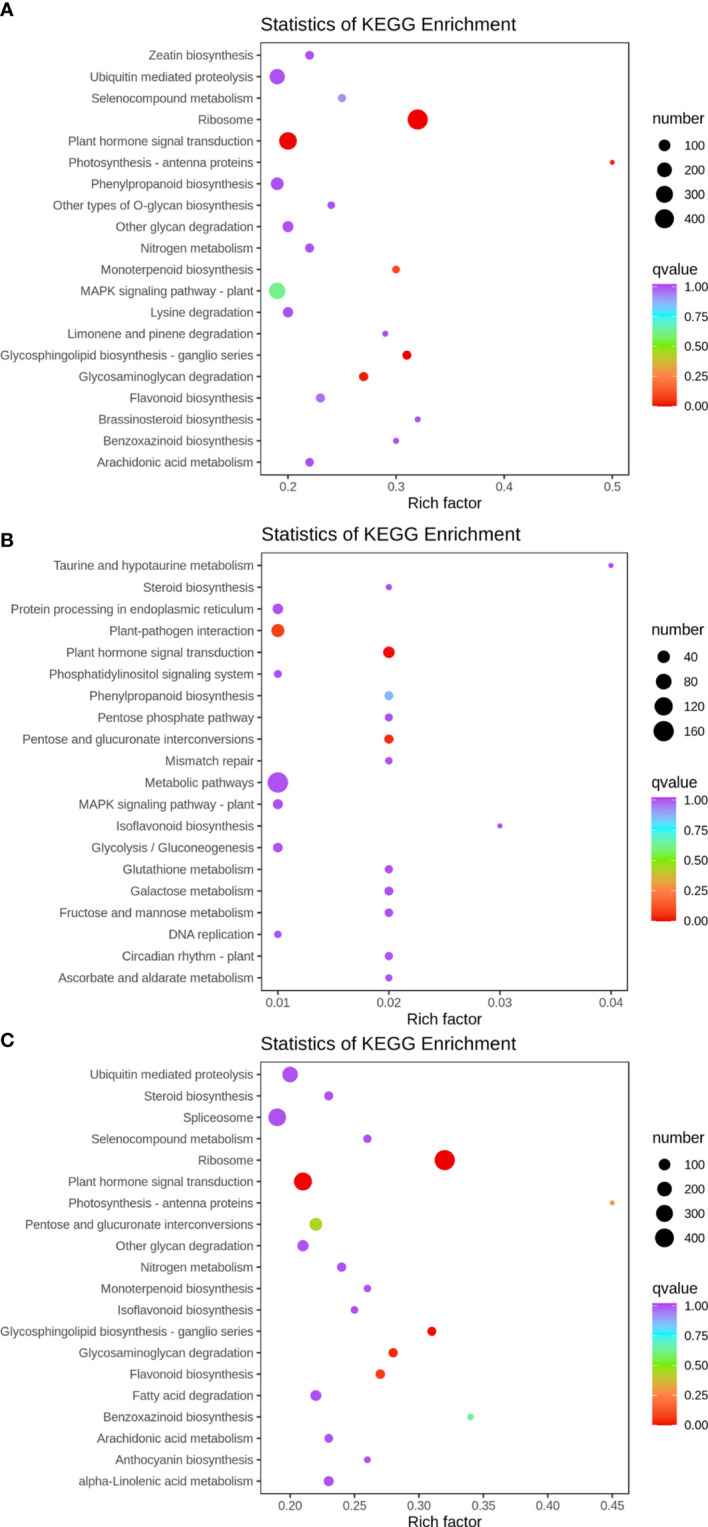
KEGG enrichment bubble plot. Differentially expressed genes generated by a comparison of **(A)** 35 d vs. 63 d, **(B)** 63 d vs. 69 d, **(C)** 35 d vs. 69 d. Rich factor represents the ratio of the number of differentially expressed genes enriched by the pathway to the number of annotated genes. The color bar represents the significance test p value adjusted for multiple hypothesis testing. The number represents the number of differentially expressed genes enriched in the pathway.

KEGG enrichment analysis was performed on the differentially expressed genes in 35 d vs. 63 d, 63 d vs. 69 d, and 35 d vs. 69 d, and the 20 pathways with the most significant enrichment were selected for representation with a bubble chart. The results are shown in **Figures 5A–C**. The plant hormone signaling pathway was enriched in all groups. Fructose and mannose metabolism, glycolysis/gluconeogenesis, and pentose phosphate pathways were found in 63 d vs. 69 d, and it was speculated that the differentially expressed genes of these pathways may have an impact on “sugar receding”.

### 3.8 Screening of differentially expressed genes

**Figure 6:**
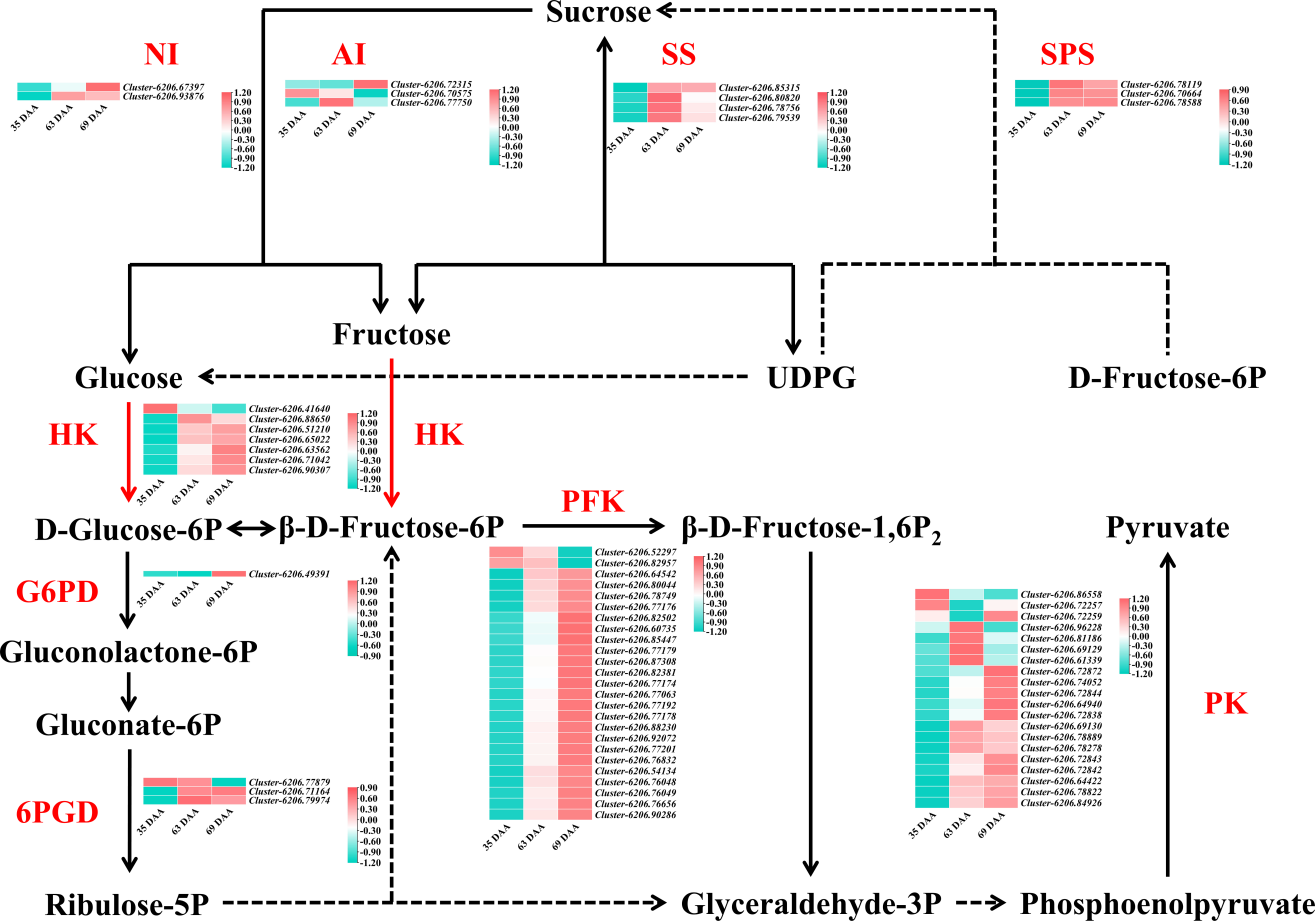
The sugar metabolism pathway map and heatmap of differentially expressed genes in 35 d vs. 63 d, 63 d vs. 69 d, 35 d vs. 69 d. The color bar represents the value of log_2_(fold change). SS, sucrose synthase; SPS, sucrose phosphate synthase; AI, acid invertase; NI, neutral invertase; HK, hexokinase; G6PD, glucose 6-phosphate dehydrogenase; 6PGD, 6-phosphogluconate dehydrogenase; PFK, phosphofructokinase; PK, pyruvate kinase.

To further analyze the differentially expressed genes related to the “sugar receding” phenomenon in ‘Feizixiao’ litchi pulp, a total of 91 genes related to sucrose metabolism, EMP and PPP were screened from the transcriptome data. No FK, SWEET and sucrose transporter (SUT) genes were screened due to low expression and no significant change in the “sugar receding” period. The log_2_(fold change) was calculated to plot the heatmap shown in **Figure 6**. In 63 d vs. 69 d, four SS genes were screened out and all were downregulated; three AI genes were screened out, of which two were downregulated; two NI genes were screened out, of which one was downregulated; three SPS genes were screened out, of which two were downregulated; seven HK genes were screened out, of which five were upregulated; Twenty-five PFK genes were screened out, of which twenty-three were upregulated; twenty PK genes were screened out, of which twelve were upregulated; one G6PD gene was screened out and it was upregulated; and three 6PGD genes were screened out, of which one was upregulated. The results showed that during the “sugar receding” period, seventy-five percent of the sucrose metabolism enzyme genes were downregulated. In EMP, seventy-seven percent of the genes were upregulated. In PPP, two genes were upregulated, and two genes were downregulated.

In summary, most of the sucrose metabolism enzyme genes were downregulated during the “sugar receding” period, most of the key genes in EMP were upregulated, and most of them were related to PFK genes. There was no significant difference in the key genes in PPP, indicating that reducing sugars may be mainly consumed through EMP and that PFK is a key rate-limiting enzyme in EMP in ‘Feizixiao’ litchi pulp.

### 3.9 Real-time PCR verification

**Figure 7:**
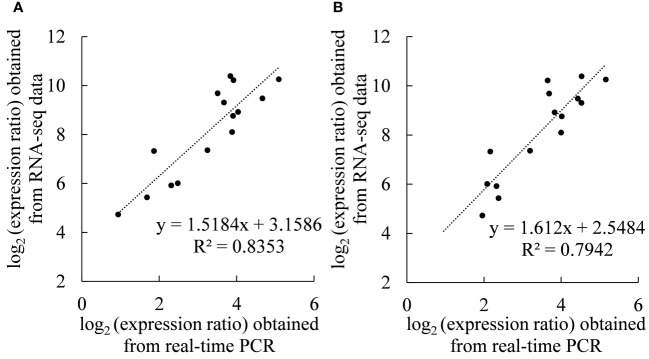
Coefficient analyses of gene expression levels obtained from the transcriptome sequencing (RNA-Seq) and real-time PCR data. Scatterplots were generated by the log_2_(expression ratios) from real-time PCR (x-axis) and RNA-seq (y-axis) in **(A)** 2020 and **(B)** 2021.

**Figures 7A, B** show that there was a significant linear correlation between the expression of 15 genes detected by real-time PCR and the transcriptome sequencing results (*r_2020_* = 0.9139, *r_2021_* = 0.8912), which proved the reliability of the transcriptome sequencing results.

## 4 Discussion

### 4.1 The relationship between sugar contents and sucrose metabolism enzyme activities

Sugar compositions and contents are key factors affecting the intrinsic quality of fruit (Borsani et al., 2009), and sucrose metabolism enzymes play an important role in sugar accumulation in fruit (Sun et al., 2020). In this study, contents of the reducing sugars in the pulp increased continuously during the growth and development process, but the content of sucrose decreased significantly at the ripening stage, resulting in an overall decrease in the total soluble sugar content (**Figures 1A, B**). Some studies have shown that AI plays an important role in sugar accumulation (Ruan, 2014). However, the activity of AI was relatively low in this study, which may suggest that AI does not play an important role in the “sugar receding” phenomenon (**Figures 2A, B**). Moreover, the activity of SS-C was consistently greater than that of SS-S (**Figures 2E–H**), and the same result was found in a study of sucrose accumulation in peaches (Vizzotto et al., 1996), which may indicate that SS plays an important role in sucrose cleavage. The activity of SS (**Figures 2E–H**) in the middle and late stages of fruit growth and development is consistent with the changes in sucrose content (**Figures 1A, B**), a previous study in citrus (*Citrus unshiu* Marc.) showed that the change in SS activity at the ripening stage was consistent with the change in total sugar content (Komatsu et al., 2002). It was speculated that SS played a greater role in the middle and late stages of fruit growth and development. The comprehensive effect of sucrose metabolism enzymes, that is, the net activity of sucrose metabolism enzymes, was an important factor affecting the sugar content of ‘Feizixiao’ litchi pulp. The reducing sugar content increased, while the activities of enzymes that cleave sucrose decreased during the “sugar receding” period (**Figures 2A, C–F**), this observation may have been related to the simultaneous decrease in the activities of enzymes that synthesize sucrose (**Figures 2G–J**), so that the net activity showed a strong sucrose cleaving activity during the “sugar receding” period (**Figures 2K, L**), resulting in a significant decrease in sucrose content (**Figures 1A, B**). Moreover, a previous study showed that sugar accumulation is related to photosynthesis (Wang et al., 2022), and the products of photosynthesis are mainly transported to the fruit in the form of sucrose. The “sugar receding” phenomenon is mainly caused by the decrease in sucrose content, and it is speculated that the photosynthetic rate of leaves may decrease during the “sugar receding” period. Therefore, to study the causes of the “sugar receding” phenomenon in ‘Feizixiao’ litchi, we should study not only the changes in sucrose metabolism enzyme activities and respiratory metabolism of pulp but also need to further study photosynthesis in leaves.

### 4.2 Analysis of the transcriptome sequencing results

**Figures 1A, B** showed that the content of sucrose in ‘Feizixiao’ litchi pulp decreased significantly, but the content of reducing sugars did not increase significantly, indicating that the downstream pathway of sugar metabolism consumes reducing sugars. The pulp of ‘Shixia’ longan (*Dimocarpus longan* Lour), belonging to the Sapindaceae family, also had the “sugar receding” phenomenon, and the research results showed that upregulated EMP, tricarboxylic acid cycle, fermentation and energy metabolism promoted “sugar receding” in the pulp (Luo et al., 2021). In a study of potato (*Solanum tuberosum* L.) tubers, it was found that low temperature reduced the activity of PFK, resulting in the inhibition of EMP, the accumulation of hexose phosphates, and ultimately an increase in sugar content (Hammond et al., 1990), indicating that PFK has a significant effect on the metabolic rate and sugar accumulation of the pulp *via* EMP. Similar results were also found in this study. The GO enrichment analysis of the differentially expressed genes in the “sugar receding” period demonstrated that PFK genes were significantly enriched in the biological process and molecular function categories (**Figure 4B**). Additionally, the KEGG enrichment analysis found that glycolysis/gluconeogenesis and pentose phosphate were significantly enriched (**Figure 5B**). A total of 91 differentially expressed genes with high expression abundance, including sucrose metabolism genes and key genes in EMP and PPP, were screened from the transcriptome sequencing data (**Figure 6**). Fifteen of these genes were selected for real-time PCR verification (**Figures 7A, B**), proving the reliability of the transcriptome sequencing results. **Figure 6** showed that the expression of SS and SPS genes was downregulated during the “sugar receding” period, which was consistent with the change in enzyme activity (**Figures 2E-J**); Seventy-seven percent of the genes associated with EMP were upregulated, while PFK genes were expressed in high abundance, and the expression of key genes in PPP showed no significant changes (**Figure 6**), indicating that reducing sugars mainly entered EMP and are consumed in large quantities and that PFK is the most critical rate-limiting enzyme in the glycolysis pathway in ‘Feizixiao’ litchi pulp. Since no sucrose transporter genes was screened out from transcriptome data, it was speculated that ‘sugar receding’ is mainly caused by the enzymes related to sucrose metabolism and EMP.

## 5 Conclusion

In summary, ‘Feizixiao’ litchi pulp mainly accumulates reducing sugars, and the reducing sugar content of the pulp remains unchanged during the fruit ripening period; however, the decrease in the sucrose content during the fruit ripening period is the main reason for the “sugar receding” phenomenon in pulp. During the fruit ripening period, the expression of PFK genes is upregulated, thereby enhancing the activity of PFK in EMP, which in turn promotes the consumption of sugar, and thereby causing the “sugar receding” phenomenon. Furthermore, the expression of sucrose metabolism genes is downregulated and the net activity of these enzymes tends to cleave sucrose, resulting in a stable reducing sugar content.

## Data availability statement

The datasets presented in this study can be found in online repositories. The names of the repository/repositories and accession number(s) can be found below: NCBI GSE218876.

## Author contributions

ZK and PJ designed the experiments. PJ, DJ, WM, CT, SX, LH, LX performed the experiments. PJ analyzed the data and wrote the manuscript. ZK revised and polished the manuscript. All authors contributed to the article and approved the submitted version.

## References

[B1] Basson C. E. Groenewald J.-H. Kossmann J. Cronjé C. Bauer R. (2010). Sugar and acid-related quality attributes and enzyme activity in strawberry fruits: Invertase is the main sucrose hydrolysing enzyme. Food Chem. 121, 1156–1162. doi: 10.1016/j.foodchem.2010.01.064

[B2] Borsani J. Budde C. O. Porrini L. Lauxmann M. A. Lombardo V. A. Murray R.. (2009). Carbon metabolism of peach fruit after harvest: Changes in enzymes involved in organic acid and sugar level modifications. J. Exp. Bot. 60, 1823–1837. doi: 10.1093/jxb/erp055 19264753

[B3] Braun D. M. Wang L. Ruan Y. L. (2014). Understanding and manipulating sucrose phloem loading, unloading, metabolism, and signalling to enhance crop yield and food security. J. Exp. Bot. 65, 1713–1735. doi: 10.1093/jxb/ert416 24347463

[B4] Castiglia D. Cardi M. Landi S. Cafasso D. Esposito S. (2015). Expression and characterization of a cytosolic glucose 6 phosphate dehydrogenase isoform from barley (*Hordeum vulgare*) roots. Protein. Expr. Purif. 112, 8–14. doi: 10.1016/j.pep.2015.03.016 25888782

[B5] Chen C. Chen H. Zhang Y. Thomas H. R. Frank M. H. He Y.. (2020). TBtools:an integrative toolkit developed for interactive analyses of big biological data. Mol. Plant 13, 1194–1202. doi: 10.1016/j.molp.2020.06.009 32585190

[B6] Granot D. David-Schwartz R. Kelly G. (2013). Hexose kinases and their role in sugar-sensing and plant development. Front. Plant Sci. 4. doi: 10.3389/fpls.2013.00044 PMC3594732 23487525

[B7] Hammond J. B. W. Burrell M. M. Kruger N. J. (1990). Effect of low temperature on the activity of phosphofructokinase from potato tubers. Planta 180, 613–616. doi: 10.1007/BF02411461 24202108

[B8] Jiang Y. Wang Y. Song L. Liu H. Lichter A. Kerdchoechuen O.. (2006). Postharvest characteristics and handling of litchi fruit — an overview. Aust. J. Exp. Agric. 46, 1541–1556. doi: 10.1071/EA05108

[B9] Jiang S. Y. Xu H. Y. Wang H. C. Hu G. B. Li J. G. Chen H. B.. (2012). A comparison of the costs of flowering in ‘Feizixiao’ and ‘Baitangying’ litchi. Sci. Hortic. 148, 118–125. doi: 10.1016/j.scienta.2012.09.035

[B10] Jiang L. Q. Ye W. W. Situ J. J. Chen Y. B. Yang X. Y. Kong G. H.. (2017). A puf RNA-binding protein encoding gene PlM90 regulates the sexual and asexual life stages of the litchi downy blight pathogen *Peronophythora litchii* . Fungal Genet. Biol. 98, 39–45. doi: 10.1016/j.fgb.2016.12.002 27939344

[B11] Kandel-Kfir M. Damari-Weissler H. German M. A. Gidoni D. Mett A. Belausov E.. (2006). Two newly identified membrane-associated and plastidic tomato HXKs: characteristics, predicted structure and intracellular localization. Planta 224, 1341–1352. doi: 10.1007/s00425-006-0318-9 16761134

[B12] Kaur G. Das N. (2022). An isoform of sucrose synthase involved in sink strength of potato (*Solanum tuberosum* l.): Molecular cloning, sequence analyses, 3-d structure, crucial motifs and expression. S AFR J. Bot. 149, 446–457. doi: 10.1016/j.sajb.2022.06.032

[B13] Koch K. (2004). Sucrose metabolism: Regulatory mechanisms and pivotal roles in sugar sensing and plant development. Curr. Opin. Plant Biol. 7, 235–246. doi: 10.1016/j.pbi.2004.03.014 15134743

[B14] Komatsu A. Moriguchi T. Koyama K. Omura M. Akihama T. (2002). Analysis of sucrose synthase genes in citrus suggests different roles and phylogenetic relationships. J. Exp. Bot. 53, 61–71. doi: 10.1093/jexbot/53.366.61 11741042

[B15] Kou J. Y. Wei Y. Y. He X. X. Xu J. Y. Xu F. Shao X. F. (2018). Infection of post-harvest peaches by monilinia fructicola accelerates sucrose decomposition and stimulates the embden–Meyerhof–Parnas pathway. Hortic. Res. 5, 46. doi: 10.1038/s41438-018-0046-x 30181886 PMC6119188

[B16] Liao H. Z. Lin X. K. Du J. J. Peng J. J. Zhou K. B. (2022). Transcriptomic analysis reveals key genes regulating organic acid synthesis and accumulation in the pulp of litchi chinensis sonn. cv. Feizixiao. Sci. Hortic. 303, 111220. doi: 10.1016/j.scienta.2022.111220

[B17] Li X. Dai L. Y. Liu H. Liu W. Pan B. L. Wang X.. (2020b). Molecular mechanisms of furanone production through the EMP and PP pathways in zygosaccharomyces rouxii with d-fructose addition. Food Res. Int. 133, 109137. doi: 10.1016/j.foodres.2020.109137 32466928

[B18] Li X. Guo W. Li J. Yue P. Bu H. Jiang J.. (2020a). Histone acetylation at the promoter for the transcription factor PuWRKY31 affects sucrose accumulation in pear fruit. Plant Physiol. 182, 2035–2046. doi: 10.1104/pp.20.00002 32047049 PMC7140945

[B19] Livak K. J. Schmittgen T. D. (2001). Analysis of relative gene expression data using real-time quantitative PCR and the 2(-delta delta C(T)) method. Methods 25, 402–408. doi: 10.1006/meth.2001.1262 11846609

[B20] Love M. I. Huber W. Anders S. (2014). Moderated estimation of fold change and dispersion for RNA-seq data with DESeq2. Genome Biol. 15, 550. doi: 10.1186/s13059-014-0550-8 25516281 PMC4302049

[B21] Luo T. Shuai L. Lai T. T. Liao L. Y. Li J. Duan Z. H.. (2021). Up-regulated glycolysis, TCA, fermentation and energy metabolism promoted the “sugar receding” in ‘Shixia’ longan (*Dimocarpus longan* lour.) pulp. Sci. Hortic. 281, 109998. doi: 10.1016/j.scienta.2021.109998

[B22] Ma Q. J. Sun M. H. Lu J. Liu Y. J. Hu D. G. Hao Y. J. (2017). Transcription factor AREB2 is involved in soluble sugar accumulation by activating sugar transporter and amylase genes. Plant Physiol. 174, 2348–2362. doi: 10.1104/pp.17.00502 28600345 PMC5543958

[B23] Menzel C. (2001). The physiology of growth and cropping in lychee. Acta Hortic. 558, 175–184. doi: 10.17660/ActaHortic.2001.558.24

[B24] Milne R. J. Grof C. P. L. Patrick J. W. (2018). Mechanisms of phloem unloading: shaped by cellular pathways, their conductances and sink function. Curr. Opin. Plant Biol. 43, 8–15. doi: 10.1016/j.pbi.2017.11.003 29248828

[B25] Pareek S. (2016). “Nutritional and biochemical composition of lychee (Litchi chinensis sonn.) cultivars,” in Nutritional composition of fruit cultivars. Ed. Simmonds M. S. J. (San Diego, FL: Academic Press), 395–418. doi: 10.1016/B978-0-12-408117-8.00017-9

[B26] Pego J. V. Smeekens S. C. M. (2000). Plant fructokinases: A sweet family get-together. Trends Plant Sci. 5, 531–536. doi: 10.1016/S1360-1385(00)01783-0 11120475

[B27] Ramos-Martinez J. I. (2017). The regulation of the pentose phosphate pathway: Remember Krebs. Arch. Biochem. Biophys. 614, 50–52. doi: 10.1016/j.abb.2016.12.012 28041936

[B28] Ren G. X. Ran X. L. Zeng R. Y. Chen J. W. Wang Y. B. Mao C. L.. (2021). Effects of sodium selenite spray on apple production, quality, and sucrose metabolism-related enzyme activity. Food Chem. 339, 127883. doi: 10.1016/j.foodchem.2020.127883 32889132

[B29] Ruan Y. L. (2014). Sucrose metabolism: gateway to diverse carbon use and sugar signaling. Annu. Rev. Plant Biol. 65, 33–67. doi: 10.1146/annurev-arplant-050213-040251 24579990

[B30] Ruan Y. L. Jin Y. Yang Y. J. Li G. J. Boyer J. S. (2010). Sugar input, metabolism, and signaling mediated by invertase: Roles in development, yield potential, and response to drought and heat. Mol. Plant 3, 942–955. doi: 10.1093/mp/ssq044 20729475

[B31] Sun L. Li C. Y. Zhu J. Jiang C. N. Li Y. H. Ge Y. H. (2020). Influences of postharvest ATP treatment on storage quality and enzyme activity in sucrose metabolism of malus domestica. Plant Physiol. Biochem. 156, 87–94. doi: 10.1016/j.plaphy.2020.09.004 32919213

[B32] Trevanion S. J. Kruger N. J. (1991). Effect of temperature on the kinetic properties of pyrophosphate: fructose 6-phosphate phosphotransferase from potato tuber. J. Plant Physiol. 137, 753–759. doi: 10.1016/S0176-1617(11)81235-6

[B33] Varet H. Brillet-Guéguen L. Coppée J.-Y. Dillies M.-A. (2016). SARTools: A DESeq2- and EdgeR-based r pipeline for comprehensive differential analysis of RNA-seq data. PLoS ONE 11, e0157022. doi: 10.1371/journal.pone.0157022 27280887 PMC4900645

[B34] Vizzotto G. Pinton R. Varanini Z. Costa G. (1996). Sucrose accumulation in developing peach fruit. Physiol. Plant 96, 225–230. doi: 10.1111/j.1399-3054.1996.tb00206.x

[B35] Wang L. Brouard E. Prodhomme D. Hilbert G. Renaud C. Petit J.. (2022). Regulation of anthocyanin and sugar accumulation in grape berry through carbon limitation and exogenous ABA application. Food Res. Int. 160, 111478. doi: 10.1016/j.foodres.2022.111478 36076369

[B36] Wang H. C. Huang H. B. Huang X. M. Hu Z. Q. (2006). Sugar and acid compositions in the arils of litchi chinensis sonn.: Cultivar differences and evidence for the absence of succinic acid. J. Hortic. Sci. Biotechnol. 81, 57–62. doi: 10.1080/14620316.2006.11512029

[B37] Wang Z. Yuan M. L. Li S. J. Gao D. Zhou K. B. (2017). Applications of magnesium affect pericarp colour in the feizixiao litchi. J. Hortic. Sci. Biotechnol. 92, 559–567. doi: 10.1080/14620316.2017.1322922

[B38] Yang Z. Y. Wang T. D. Wang H. C. Huang X. M. Qin Y. H. Hu G. B. (2013). Patterns of enzyme activity and gene expressions in sucrose metabolism in relation to sugar accumulation and composition in the aril of litchi chinensis sonn. J. Plant Physiol. 170, 731–740. doi: 10.1016/j.jplph.2012.12.021 23499454

[B39] Zhang Y. X. Yu D. Liu C. Y. Gai S. P. (2018). Dynamic of carbohydrate metabolism and the related genes highlights PPP pathway activation during chilling induced bud dormancy release in tree peony (*Paeonia suffruticosa*). Sci. Hortic. 242, 36–43. doi: 10.1016/j.scienta.2018.07.022

[B40] Zhao Y. Y. Tang J. X. Brummell D. A. Song C. C. Qi S. N. Lin Q.. (2022). Abscisic acid alleviates chilling injury in cold-stored peach fruit by regulating the metabolism of sucrose. Sci. Hortic. 298, 111000. doi: 10.1016/j.scienta.2022.111000 PMC9488807 36147223

