# Supplementary material for: Transcriptomics‐based analysis of the causes of sugar receding in Feizixiao litchi (Litchi chinensis Sonn.) pulp
Source: Front Plant Sci. 2022 Dec 22;13:1083753. doi: 10.3389/fpls.2022.1083753 (PMC9814114; doi:10.3389/fpls.2022.1083753)
Supplement: Supplementary file 1 [file Table_1.docx]

**Supplementary Table S1. Internal reference and real-time PCR primers.**

| Gene ID | Forward primer (5’ to 3’) | Reverse primer (5’ to 3’) |
| --- | --- | --- |
| *Actin* | AGTTTGGTTGATGTGGGAGAC | TGGCTGAACCCGAGATGAT |
| *Cluster-6206.78756* | CTGCCTCCATTTGTGGCTAT | CAAAATCAAGCTCGAGCACA |
| *Cluster-6206.70664* | AGCGCAAACTGAGAGCTAGG | AGACCAAATCGGTGGATCAG |
| *Cluster-6206.78119* | ACGAGCTAGGATCAGGCGTA | AAGAGGGGTGGTCTTCGTTT |
| *Cluster-6206.78749* | GGGCTGTTTGAATTCCTTGA | CTCTTCCTTCTGGGCATCAG |
| *Cluster-6206.85447* | CATGATCCGTGCTATTCCAA | GTACCCAGCCATAGCACCAT |
| *Cluster-6206.77179* | GCAATGCATCCGATAATGTG | AACAGGGCCAACTGTGAAAC |
| *Cluster-6206.77063* | CGAGAATGGTGTTGGTGTTG | GTCCACCAGGTCCTTCAAGA |
| *Cluster-6206.88230* | GTCGCCCAAAAGATTGTTGT | GCAAAGACCACCACACGTTA |
| *Cluster-6206.82381* | GCATTGCATCCGATAATGTG | GGACCAACTGTGAAGCCTGT |
| *Cluster-6206.77201* | TGACATCCATAAGCGTGGTG | AGCCCCTTTCTGAGTTCCAT |
| *Cluster-6206.77192* | ACCCCACCTTCTCTTGCTCT | GGAGTTCTCGGAAATGACCA |
| *Cluster-6206.77174* | GCAATGCATCCGATAATGTG | ACCCGATGGAACGGTATGTA |
| *Cluster-6206.82502* | TCGGAGGAGATGGAACTCAG | ATTCCAGCCACAGCAACTTT |
| *Cluster-6206.72872* | CCAAGGGAAGCCTGTTGTAA | TCAGTGCCATCAAGAACTGC |
| *Cluster-6206.74052* | TTGGTTCCGGCTTTATATGC | TTGCAGAATCCCTTGCTCTT |
