# Supplementary material for: Transcriptomics‐based analysis of the causes of sugar receding in Feizixiao litchi (Litchi chinensis Sonn.) pulp
Source: Front Plant Sci. 2022 Dec 22;13:1083753. doi: 10.3389/fpls.2022.1083753 (PMC9814114; doi:10.3389/fpls.2022.1083753)
Supplement: Supplementary file 2 [file Table_2.docx]

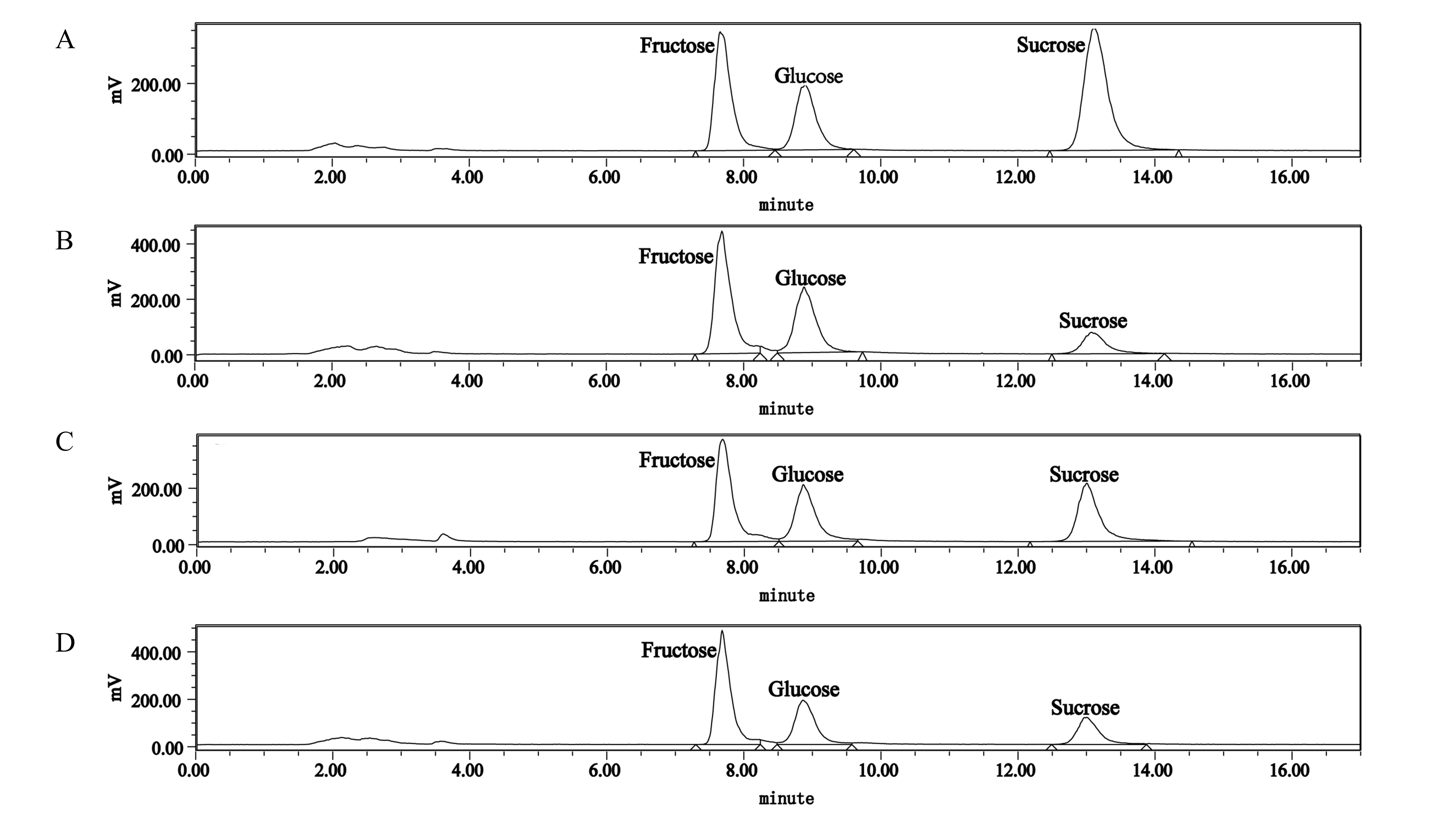
**Supplementary Figure S1.** HPLC chromatograms of sugar components in ‘Feizixiao’ litchi pulp. (A) (B) HPLC chromatograms of sugar contents in fruit pulp at 63 and 69 DAA in 2020. (C) (D) HPLC chromatograms of sugar contents in fruit pulp at 69 and 70 DAA in 2021.
